# Clinical and prognostic significance of HIF-1α overexpression in oral squamous cell carcinoma: a meta-analysis

**DOI:** 10.1186/s12957-017-1163-y

**Published:** 2017-05-18

**Authors:** Jianhua Zhou, Shengyun Huang, Lili Wang, Xiao Yuan, Quanjiang Dong, Dongsheng Zhang, Xuxia Wang

**Affiliations:** 1 Department of Oral and Maxillofacial Surgery, School of Stomatology, Shandong University, Jinan, Shandong 250012 China; 2 Department of Oral and Maxillofacial Surgery, Shandong Provincial Hospital, Affiliated to Shandong University, Jinan, Shandong 250012 China; 3 Department of Stomatology, Qingdao Municipal Hospital, Qingdao, Shandong 266071 China; 4 Central laboratories, Qingdao Municipal Hospital, Qingdao, Shandong 266071 China

**Keywords:** Oral squamous cell carcinoma, Prognosis, Hypoxia inducible factor-1α, Meta-analysis

## Abstract

**Background:**

Recent studies have indicated an association between hypoxia inducible factor-1 alpha (HIF-1α) expression and poor prognosis in patients with oral squamous cell carcinoma (OSCC); however, definitive evidence of this association is yet to be obtained. We performed a meta-analysis to evaluate the association of HIF-1α expression with clinicopathological characteristics and overall survival (OS) of patients with OSCC.

**Methods:**

A literature search for relevant studies published in English language as of February 05, 2016, was performed on PubMed, Web of Science, and EMBASE databases. Eighteen studies with a combined study population of 1474 patients with OSCC are included in the meta-analysis. Odds ratio (OR) or hazard ratio (HR) with 95% confidence interval (CI) was calculated using random-effects model or fixed-effects model.

**Results:**

HIF-1α overexpression was significantly associated with larger tumor size (OR = 2.28, 95% CI = 1.49–3.50, *P* = 0.017), advanced TNM stage (OR = 2.29, 95% CI = 1.50–3.49, *P* = 0.158), and lymph node metastasis (OR = 2.05, 95% CI = 1.19–3.53, *P* < 0.001), but not with poor differentiation (OR = 1.21, 95% CI = 0.55–2.64, *P* = 0.024). These results demonstrated an association between HIF-1α expression and biological behavior of OSCC. On pooled analyses, high expression of HIF-1α was associated with worse OS (HR = 1.70, 95% CI = 1.10–2.61, *P* < 0.001). On subgroup analyses, overexpression of HIF-1α was significantly associated with poor prognosis in Asian population (HR = 2.33, 95% CI = 1.72–3.15, *P* = 0.862).

**Conclusions:**

Our findings demonstrate an association of HIF-1α overexpression with tumor size, tumor stage, lymph node metastasis, and overall survival. HIF-1α could be an independent prognostic marker in patients with OSCC.

## Background

Oral squamous cell carcinoma shows a high propensity for invasive growth and cervical lymph node metastasis [1]. Every year, an estimated 263,000 new cases of oral squamous cell carcinoma (OSCC) are reported worldwide [2]. Five-year survival rates of patients with OSCC have remained in the vicinity of 50% over the last few decades [3, 4].

Solid malignant tumors tend to show rapid proliferation and growth in a hypoxic microenvironment [5]. Hypoxia inducible factor-1 (HIF-1) is an important regulator of cellular response to hypoxia. It is a heterodimer that comprises of HIF-1α and HIF-1β subunits [5–7], the former being its functional subunit [8]. High expression levels of HIF-1α, a marker of tumor hypoxia, have been reported in patients with OSCC [9]. Overexpression of HIF-1α has recently been linked with unfavorable prognosis in several types of malignant tumors [10, 11]. However, definitive evidence of this association is yet to be obtained.

Though a previous meta-analysis revealed an association between HIF-1α expression and poor overall survival in patients with OSCC, the analysis was not comprehensive [12]. The main prognostic factors for OSCC include tumor size, lymph node status, tumor stage, and differentiation. We performed a literature review to evaluate the association of HIF-1α expression with clinicopathological characteristics and overall survival in patients with OSCC.

## Methods

### Search strategy

Full-text original research articles relating to expression of HIF-1α in patients with OSCC were identified on a search of PubMed, Web of Science, and EMBASE databases on February 05, 2016. The following keywords were used: “hypoxia inducible factor 1α,” “HIF 1α,” “oral squamous cell carcinoma,” “OSCC,” “mouth neoplasm,” “oral cancer,” “oral carcinoma,” or “oral tumor” with all possible combinations.

The inclusion criteria were as follows: (1) Language of publication: English; (2) studies that investigated the correlation between HIF-1α and prognosis in patients with a histopathological diagnosis of squamous cell carcinoma; (3) HIF-1α protein expression determined by immunohistochemical examination of paraffin-embedded surgical specimens; and (4) adequate data for estimation of hazard ratio and overall survival reported.

Conference abstracts, reviews, letters, case reports, unpublished studies, duplicate publications, and experimental studies were excluded from the analysis.

### Data extraction

Two researchers independently reviewed the eligible studies and performed data extraction using a standard format. Data pertaining to the following variables were extracted: name of first author, year of publication, country, sex distribution, sample size, definition of HIF-1α positive, clinicopathological features, and overall survival analysis. Any disagreement on the eligibility criteria was resolved by consensus among the authors.

### Methodological assessment

The Newcastle-Ottawa quality assessment scale (NOS) was used for methodological assessment of the included studies [13]. The maximum score was 9; studies with a total score of ≥6 points were deemed to be of a high quality. Two researchers assessed the scores independently; any disagreements in this respect were resolved by consensus.

### Statistical analyses

Statistical analyses were performed using Stata (version 11.0; Stata Corporation, College Station, Texas, USA). Kaplan-Meier curves were plotted using Engauge Digitizer version 4.1 (free software downloaded from http://sourceforge.net). Hazard ratio at 95% confidence interval was used to assess the relationship between HIF-1α and overall survival. An HR >1 correlated with poor prognosis. HRs were either estimated directly from the reported data using methods described by Parmar et al. [14] or were derived from the Kaplan-Meier curve. Odds ratio at 95% CI were calculated to evaluate the association of HIF-1α expression with clinicopathological features.

Heterogeneity of the included studies was checked by chi-square-based Q statistical test. A value of *I* ^2^ < 50% or *P* value >0.1 was considered to be indicative of no significant heterogeneity, and a fixed-effects model was used for analysis. Otherwise, the random-effects model was used.

Sensitivity analysis was performed to evaluate the robustness of the individual studies. Begg’s funnel plot and Egger’s regression test were used to evaluate the effect of potential publication bias on the results of this analysis [15]. All *P* values were two-sided; *P* < 0.05 was considered as statistically significant.

## Results

### Study characteristics

**Fig. 1:**
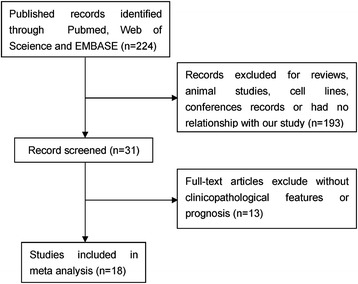
Schematic illustration of the literature search and study selection criteria

As shown in Fig. 1, a total of 224 studies were retrieved on initial literature search. After scanning the titles, abstracts, and full texts, 18 studies [5, 6, 16–30] were finally included in the meta-analysis. All studies were retrospective analytic studies and were published between 2005 and 2015. Out of the 18 studies, 15 had assessed the relationship between HIF-1α expression and clinicopathological features, and 14 studies had reported survival data directly or indirectly. The sample sizes ranged from 25 to 233 cases (mean sample size, 82). The combined patient population was 1474. These included 561 patients with well- or moderately differentiated tumors, and 182 patients with poorly differentiated tumors. A total of 159 patients were categorized as clinical stage I/II disease and 264 as clinical stage III/IV disease; 539 patients were graded as T1/T2 and 531 patients as T3/T4. A total of 610 patients had lymph node involvement, while no lymph node involvement was observed in 605 patients. These studies were completed in six countries (Brazil, Korea, China, Japan, Germany, and Australia).

Although the expression of HIF-1α was determined on immunohistochemistry (IHC), the procedure varied between different studies. Table 1 shows the main characteristics and quality of these 18 included studies.

**Table 1:** Main characteristics and quality of the studies included in the meta-analysis

First author	Year	Country	Gender (male/female)	Number	Criteria used for high HIF-1α expression	Clinical features	OS analysis	Quality evaluation			
								Selection	Comparability	Outcome	Total points
Lin	2008	China, Taiwan	54/3	57	>60%	NR	Reported	3	0	2	5
Liang	2011	China	49/40	89	>25%	T/LN/D	Reported	3	1	3	7
Huang	2012	China	46/34	80	>4^a^	LN	Reported	2	1	3	6
Han	2012	Korea	20/13	33	>10%	NR	Reported	4	1	2	7
Zheng	2013	China	66/54	120	>5%	T/LN/D	Reported	4	1	2	7
Hwa	2015	Korea	17/8	25	>50%	NR	Reported	3	1	2	6
Zhu	2010	China	52/45	97	Positive staining	T/LN/D	Reported	3	1	3	7
Kang	2013	China	28/21	49	>10%	T/LN	Reported	4	1	3	8
Hong	2013	Australia	186/47	233	>10%	T/LN/D	Reported	4	1	2	7
Eckert	2010	Germany	60/20	80	>6^a^	T/LN/D/TNM	Reported	3	1	2	6
Fillies	2005	Germany	71/14	85	>5%	NR	Reported	2	0	2	4
Santos	2012	Brazil	56/10	66	>6^a^	T/LN/D/TNM	Reported	4	0	3	7
Mendes	2014	Brazil	48/8	56	>3^a^	T/LN/D/TNM	Reported	3	0	2	5
Eckert	2011	Germany	60/22	82	>6^a^	T/LN/D/TNM	Reported	2	1	3	6
Liang	2008	China	40/25	65	>10%	LN/TNM	NR	3	1	2	6
Naruse	2011	Japan	72/48	120	>4^a^	T/LN	NR	3	0	2	5
Zhang	2014	China	52/28	80	>4^a^	T/LN/D	NR	3	1	1	5
Vasconcelos	2015	Brazil	40/17	57	>10%	TNM	NR	2	1	1	4

*NR* not reported, *T* tumor size, *LN* lymph node status, *TNM* TNM stage, *D* differentiation

^a^The final score for samples was calculated based on a combination of percentage of stained cells and staining intensity

### Association of HIF-1α expression with clinicopathological characteristics

**Fig. 2:**
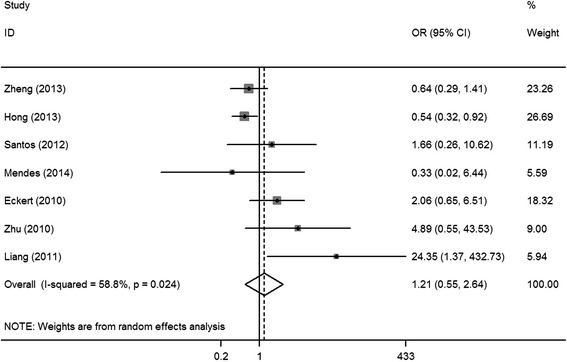
Forest plot of the association between HIF-1α expression and histological differentiation of OSCC

**Fig. 3:**
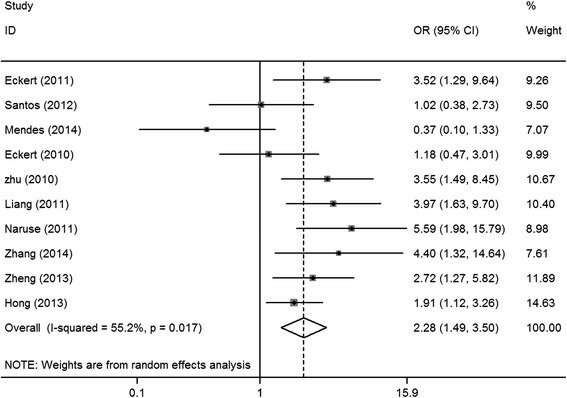
Forest plot of the association between HIF-1α expression and tumor size

**Fig. 4:**
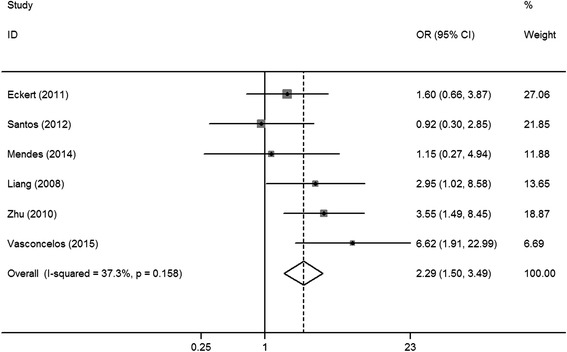
Forest plot of the association between HIF-1α expression and TNM stage

Seven studies [8, 16–21] had reported on the association of HIF-1α expression with tumor histology. High HIF-1α expression did not show any significant association with poor histological differentiation (OR = 1.21, 95% CI = 0.55–2.64, *I* ^2^ = 58.8%, *P* = 0.024; random-effects model) (Fig. 2). Ten studies [6, 8, 16–23] had assessed the association between HIF-1α expression and tumor size (T3/T4 vs. T1/T2). One study was excluded from the analysis owing to inconsistent cut-off values for tumor size (T3/T2 vs. tumor in situ) [24]. Owing to significant heterogeneity among the included studies, a random-effects model was applied in this meta-analysis. Pooled OR was 2.28 (95% CI = 1.49–3.50, *I* ^2^ = 55.2%, *P* = 0.017; random-effects model) (Fig. 3), which suggested an association between HIF-1α expression and tumor size. In six studies [6, 19–21, 25, 26], patients with stage III/IV OSCC had higher levels of HIF-1α expression as compared to that in patients with stage I/II tumors. The combined OR was significant (OR = 2.29, 95% CI = 1.50–3.49, *I* ^2^ = 37.3%, *P* = 0.158; fixed-effects model) with no significant heterogeneity (Fig. 4).

**Fig. 5:**
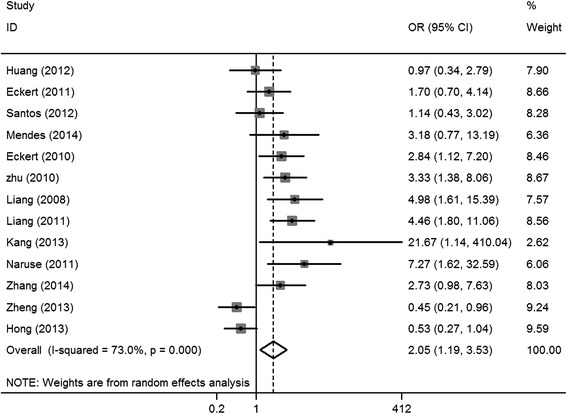
Forest plot of the association between HIF-1α expression and lymph node status

A significant association between HIF-1α expression and lymph node status was reported in 13 studies [6, 8, 16–25, 28] (OR = 2.05, 95% CI = 1.19–3.53, *I* ^2^ = 73%, *P* < 0.001; random-effects model); significant heterogeneity was observed among these studies (Fig. 5).

### Overall survival correlated with HIF-1α overexpression

A total of fourteen studies (combined *N* = 1152) had evaluated the association of HIF-1α expression with overall survival. One study, for which the Kaplan-Meier curve or the original data was not available, was excluded from the meta-analysis of OS [23]. A random-effects model was performed for the calculation of HRs owing to significant heterogeneity among these studies (*χ* ^2^ = 46.26, *P* < 0.0001, *I* ^2^ = 71.9%).

**Fig. 6:**
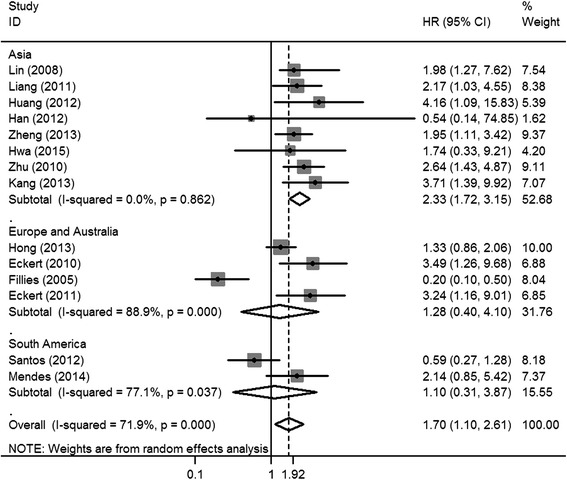
Forest plot of the association between HIF-1α expression and overall survival

The pooled HR for the HIF-1α (+) vs. HIF-1α (−) group was 1.70 (95% CI = 1.10–2.61) (Fig. 6). On subgroup analysis, high HIF-1α expression significantly correlated with poorer prognosis in Asian patients with OSCC (HR = 2.33, 95% CI = 1.72–3.15); there was no significant heterogeneity in this respect (*I* ^2^ = 0.0%, *P* = 0.862) (Fig. 6).

### Sensitivity analysis and publication bias

**Fig. 7:**
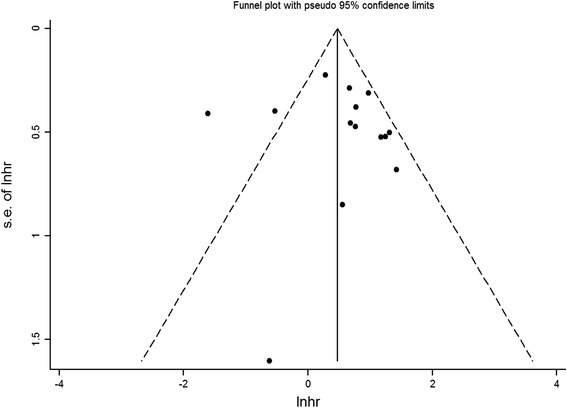
Begg’s funnel plot for evaluation of publication bias

Sensitivity analysis was performed to evaluate the robustness of the analysis. Pooled HR was not significantly influenced by any single study, as indicated by analysis performed after a sequential omission of one study at a time (data not shown). Both Begg’s (Fig. 7) and Egger’s (data not shown) tests indicated a lack of significant publication bias (*P* > 0.05).

## Discussion

Oral squamous cell carcinoma is a fatal disease; the typically poor prognosis is attributable to its invasive growth. Surgical resection is the mainstay of treatment. Nonetheless, survival rates of patients with OSCC have not shown improvement over the last few decades. More reliable therapeutic biomarkers are urgently needed for the treatment of OSCC. The results of our meta-analysis showed an association of HIF-1α expression levels with clinicopathological characteristics and overall survival of these patients.

HIF-1α degradation is inhibited under hypoxic conditions as well as under some conditions also under normoxic conditions [31]. HIF-1α activates the transcription of downstream target genes which regulate many biological processes, including angiogenesis, cell proliferation, glucose metabolism, pH regulation, and migration [12, 32]. Overexpression of HIF-1α has been shown to be associated with tumor cell growth in patients with cervical, endometrial, gastric, colorectal, pancreatic, ovarian, breast, and head/neck carcinomas [33–37]. However, a consensus on this association is yet to be attained.

Zhu et al. reported a significant association of HIF-1α overexpression with tumor stage, histological differentiation, lymph node status, and poor prognosis [21]. In contrast, Fillies et al. reported significantly lower OS in patients with low expression of HIF-1α [27]. Therefore, we performed this meta-analysis to determine the clinical and prognostic significance of HIF-1α overexpression in patients with OSCC.

The results of the present meta-analysis showed that increased expression of HIF-1α protein, as detected with IHC, was significantly associated with larger tumor size (T3/T4), more advanced TNM stage (III/IV), and lymph node metastasis, but not with poor differentiation. This suggested an association of HIF-1α overexpression with the biological behavior of OSCC. High expression of HIF-1α was defined based on a combination of the percentage of stained cells and the intensity of staining. Heterogeneity in tumor size, lymph node status, and histology were eliminated. We found this method as being particularly useful to define HIF-1α expression in OSCC cells.

In the present meta-analysis, 14 eligible studies were included for the assessment of the association of HIF-1α and overall survival. The results showed a significant association between HIF-1α overexpression and poor overall survival. On subgroup analyses, a significant association between HIF-1α overexpression and poor prognosis was found only in Asian population (HR = 2.33, 95% CI = 1.72–3.15), without significant heterogeneity in this respect. Therefore, this association is subject to considerable geographical variability. It is pertinent to mention here that geographical variations have been reported in the context of gastric cancer and head-neck cancer [32, 38]. This phenomenon may be attributable to genetic differences between different ethnic groups [39]. Further studies are required to clarify this issue.

Some limitations of the present study need to be considered while interpreting our results. Firstly, only studies published in English were included in this analysis, which may have introduced an element of publication bias. Secondly, preoperative chemoradiation may have affected the prognosis of patients with OSCC. Data on preoperative treatment were available only for six studies, which may have contributed to the heterogeneity. Thirdly, the number of eligible studies was relatively small, which limits the statistical powder of our analysis. Finally, HRs were extracted from Kaplan-Meier curves in a few studies, which may not have been entirely accurate, thus contributing to the heterogeneity.

## Conclusions

In conclusion, this meta-analysis provides a strong evidence of the association of HIF-1α overexpression with both clinicopathological features and overall survival in patients with OSCC. Overexpression of HIF-1α was significantly correlated with poorer prognosis in Asian population. HIF-1α appears to be a valuable prognostic biomarker in patients with OSCC.

## Abbreviations

CI: Confidence interval
HIF-1α: Hypoxia inducible factor-1 alpha
HR: Hazard ratio
IHC: Immunohistochemistry
NOS: Newcastle-Ottawa quality assessment scale
OR: Odds ratio
OS: Overall survival
OSCC: Oral squamous cell carcinoma
